# Routes of Hendra Virus Excretion in Naturally-Infected Flying-Foxes: Implications for Viral Transmission and Spillover Risk

**DOI:** 10.1371/journal.pone.0140670

**Published:** 2015-10-15

**Authors:** Daniel Edson, Hume Field, Lee McMichael, Miranda Vidgen, Lauren Goldspink, Alice Broos, Deb Melville, Joanna Kristoffersen, Carol de Jong, Amanda McLaughlin, Rodney Davis, Nina Kung, David Jordan, Peter Kirkland, Craig Smith

**Affiliations:** 1 Queensland Centre for Emerging Infectious Diseases, Department of Agriculture and Fisheries, Coopers Plains, Queensland, Australia; 2 EcoHealth Alliance, New York, New York, United States of America; 3 Elizabeth Macarthur Agricultural Institute, New South Wales Department of Primary Industries, Menangle, NSW, Australia; 4 Wollongbar Primary Industries Institute, New South Wales Department of Primary Industries, Wollongbar, NSW, Australia; Metabiota, UNITED STATES

## Abstract

Pteropid bats or flying-foxes (*Chiroptera*: *Pteropodidae*) are the natural host of Hendra virus (HeV) which sporadically causes fatal disease in horses and humans in eastern Australia. While there is strong evidence that urine is an important infectious medium that likely drives bat to bat transmission and bat to horse transmission, there is uncertainty about the relative importance of alternative routes of excretion such as nasal and oral secretions, and faeces. Identifying the potential routes of HeV excretion in flying-foxes is important to effectively mitigate equine exposure risk at the bat-horse interface, and in determining transmission rates in host-pathogen models. The aim of this study was to identify the major routes of HeV excretion in naturally infected flying-foxes, and secondarily, to identify between-species variation in excretion prevalence. A total of 2840 flying-foxes from three of the four Australian mainland species (*Pteropus alecto*, *P*. *poliocephalus* and *P*. *scapulatus*) were captured and sampled at multiple roost locations in the eastern states of Queensland and New South Wales between 2012 and 2014. A range of biological samples (urine and serum, and urogenital, nasal, oral and rectal swabs) were collected from anaesthetized bats, and tested for HeV RNA using a qRT-PCR assay targeting the M gene. Forty-two *P*. *alecto* (n = 1410) had HeV RNA detected in at least one sample, and yielded a total of 78 positive samples, at an overall detection rate of 1.76% across all samples tested in this species (78/4436). The rate of detection, and the amount of viral RNA, was highest in urine samples (>serum, packed haemocytes >faecal >nasal >oral), identifying urine as the most plausible source of infection for flying-foxes and for horses. Detection in a urine sample was more efficient than detection in urogenital swabs, identifying the former as the preferred diagnostic sample. The detection of HeV RNA in serum is consistent with haematogenous spread, and with hypothesised latency and recrudesence in flying-foxes. There were no detections in *P*. *poliocephalus* (n = 1168 animals; n = 2958 samples) or *P*. *scapulatus* (n = 262 animals; n = 985 samples), suggesting (consistent with other recent studies) that these species are epidemiologically less important than *P*. *alecto* in HeV infection dynamics. The study is unprecedented in terms of the individual animal approach, the large sample size, and the use of a molecular assay to directly determine infection status. These features provide a high level of confidence in the veracity of our findings, and a sound basis from which to more precisely target equine risk mitigation strategies.

## Introduction

Hendra virus (HeV) is a paramyxovirus of the genus *Henipavirus* first described in September 1994 in Australia, when it was identified as the aetiological agent in an outbreak of acute equine respiratory disease that resulted in the deaths of 14 horses and 1 human, in the Brisbane suburb of Hendra [1]. The subsequent retrospective identification of two equine cases in northern Queensland a month earlier prompted an extensive serological survey of Queensland horses which found no evidence of other previous outbreaks. Serologic screening of a limited number of other domestic animals (e.g. cattle, dogs, cats), rodents and native terrestrial wildlife species also found no evidence of infection [2]. The focus then shifted to Australian flying-foxes (*Chiroptera*: *Pteropodida*e), which were identified as the likely primary wildlife reservoir through a series of serological and viral isolation studies [3–5]. Antibodies were detected in all four species of Australian mainland flying-foxes, namely the Black flying-fox (*P*. *alecto*), Grey-headed flying-fox (*P*. *poliocephalus*), Spectacled flying-fox (*P*. *conspicillatus*) and Little red flying-fox (*P*. *scapulatus*) [4]. To 31 July 2015, there have been 52 reported equine incidents involving 94 confirmed or possible equine cases, and 7 associated human cases, with case fatality rates approaching 90% and 60% respectively [6].

Initial experimental HeV transmission studies with *P*. *poliocephalus* yielded inconsistent results; most infected animals seroconverted, but positive immunohistochemistry (IHC) findings were rarely observed, and virus isolation was mostly unsuccessful in a diverse range of tissues [7, 8]. In one of the two pregnant female *P*. *poliocephalus* where live virus was recovered, vertical transmission was also demonstrated by the isolation of virus from the foetal spleen and kidney [8]. Vertical transmission was also a feature when Hendra virus was isolated for the first time from two naturally-infected flying-foxes, including the uterine fluid and pooled foetal lung-liver of a pregnant *P*. *poliocephalus*, and from the foetal lung of a pregnant *P*. *alecto* [3]. A more recent experimental infection study utilising *P*. *alecto* essentially replicated earlier findings, with only 10 of the 20 HeV-inoculated animals seroconverting, negative IHC results in all tissues, and little evidence of HeV infection on histopathological examination of tissues post-mortem [9]. Halpin et al. (2011) did however detect HeV RNA by qRT-PCR in a limited range of tissues, swabs, blood and urine samples collected from the 10 animals that seroconverted within the study period. Live virus was also isolated from the urine of a single *P*. *alecto* on days 6 and 8 post-inoculation, albeit at low titres at the limit of detection, and with no evidence of HeV antigen on IHC staining in either the bladder or kidney post-mortem [9]. All experimental and natural infection studies to date support the hypothesis that HeV infection in flying-foxes is largely subclinical in nature [10].

While the apparent lack of clinical disease attributable to HeV infection in flying-foxes is consistent with coevolution theories pertaining to intimate host-pathogen relationships [11, 12], the inability to routinely recover virus from either naturally or experimentally-infected flying-foxes is problematic from an epidemiological perspective. The inherent difficulties in isolating HeV from experimentally infected flying-foxes, and the unusually low levels of virus observed in animals where infectious material is recovered, has led to the development of several novel hypotheses in terms of both HeV transmission between flying-foxes, and from flying-foxes to horses. For example, Halpin et al. (2011) conclude “that the opportunity for [equine] spillover of HeV from healthy bats is limited by the amount of excreted virus and the time over which it is excreted”, while Plowright et al. (2015) put forward the idea of cumulative exposure to small, incremental doses of infectious HeV material within a flying-fox roost that would progressively lead to more and more individual bats becoming infected over time, with the potential to trigger an epidemic [9, 13]. Daniels et al. (2007) speculated that perhaps an intermediary host that is highly susceptible to infection, capable of generating high levels of infectious virus, and distributed throughout the range of both flying-foxes and horses is required to facilitate effective spillover to horses, and suggested that domestic cats might fulfil these criteria [14]. Ideas such as these however, are largely predicated on the notion that the average flying-fox is relatively resistant to infection, and once infected sheds very low levels of virus, as observed in experimental infection studies.

The problem with extrapolating the results of experimental HeV infection studies to likely modes of transmission between wild flying-foxes, or indeed from wild flying-foxes to equine spillover hosts, is the extent to which experimental infection models truly reflect the biology of HeV in naturally-infected flying-foxes. While experimental infection studies can yield useful information, they are often subject to important caveats (e.g. artificial routes of exposure; very large infectious doses; small sample sizes; species selection; sex and age classes of animals studied; limited use of control cohorts), and their direct relevance to gaining an accurate understanding of natural disease systems may be questionable [15]. Advancements in molecular diagnostic techniques, utilising more sensitive tests such as qRT-PCR, have facilitated the study of natural HeV infection in wild flying-fox populations. In contrast to the aforementioned experimental studies [7–9], HeV RNA has been routinely detected in pooled urine samples collected beneath flying-fox roosts using qRT-PCR, and in addition four HeV isolates have been cultured from a subset of these HeV-positive pooled urine samples [16, 17]. Other novel bat paramyxoviruses have also been successfully isolated from this collection of pooled urine samples, including a novel Henipavirus (Cedar virus), and several novel Rubulaviruses, including Menangle virus [18–20]. Vidgen et al. (2015) have also identified a large number (n = 31) of unidentified flying-fox paramyxoviruses in pooled urine samples collected between 2010–2013, using a degenerative RT-PCR specific for the L-gene of the Henipavirus, Respirovirus and Morbillivirus genera [21]. Collectively these results suggest that flying-fox urine is likely to be an important route of excretion for a diverse range of paramyxoviruses.

The importance of understanding the main routes of HeV excretion in naturally-infected flying-foxes can be viewed on a number of levels. While we have strong evidence that urine is an important infectious medium that likely drives bat to bat transmission, and in all likelihood bat to horse transmission, we are less certain about the relative importance of alternative routes of excretion (such as nasal and oral secretions, and faeces), notwithstanding a small number of detections in oral and rectal swabs in both experimentally and naturally-infected *P*. *alecto* [9, 22]. If, for example, HeV is routinely excreted in nasal secretions, then aerosolised dissemination of virus becomes more likely, which may favour highly effective, density-dependent bat to bat transmission within the typically dense, three-dimensional aggregations of individual bats characteristic of Australian flying-fox roosts. Alternatively, oral excretion of the virus would require closer social contact for successful virus transmission, and frequency-dependent interactions with conspecifics such as allogrooming, territorial aggression, or mating behaviour could impart strong spatiotemporal structures on viral infection dynamics. Differentiating between alternative modes of transmission is critical in determining transmission rates in host-pathogen models [23], and while previous HeV models have attempted to parameterise transmission rates both within and between roosts [24], they would almost certainly be improved by empirical data on relative infection rates in different bat excreta. Determining the main routes of HeV excretion in flying-foxes is also important when assessing the relative risk of spillover to horses on putative exposure to flying-fox derived infectious material.

The primary aim of this study was to determine the major routes of HeV excretion in flying-foxes. A secondary aim was to identify any between-species variation in excretion prevalence. The expectation is that the findings will more precisely inform equine risk mitigation strategies and add to our understanding of the relative risk of HeV infection at the flying-fox species level.

## Methods

### Ethics statement

Fieldwork in all locations was approved under the Queensland Department of Agriculture, Fisheries and Forestry Animal Ethics Committee Permit SA 2011/12/375 and the Queensland Department of Environment, Heritage and Protection Scientific Purposes Permits WISP05810609 and WISP14100614. Full details of capture and sampling methods are presented below. All methods represent current best practice and were specifically approved under the permits. Capture and sampling was undertaken by trained and experienced teams including a veterinarian. No animals were sacrificed.

### Study design and sampling locations

Flying-foxes were sampled at multiple roost locations in the eastern Australian states of Queensland (QLD) and New South Wales (NSW) between February 2012 and June 2014. The study utilised capture events associated with two related studies: a cross-sectional study series investigating the association between flying-fox roost disturbance and HeV infection dynamics [25]; and a structured longitudinal study investigating HeV infection and transmission dynamics in flying-foxes [26]. The study objectives and the data analysis are independent of these other studies.

### Capture and sampling

Flying-foxes were captured pre-dawn, as they returned to the roost, in 18 m mist nets suspended between either 12 m aluminium poles or 20 m fibreglass telescopic poles (Spiderbeam GmbH). Animals were carefully extracted from the mist net and immediately placed in a pillow-case tied to a horizontal line, which enabled bats to roost in a natural inverted position while awaiting sampling. All flying-foxes were anesthetised for the collection of samples, as described by Jonsson et al. (2004) [27]. Species, sex, age class, reproductive status, weight, forearm length and body condition score were recorded for each individual bat. Criteria for each of these classification schemes are detailed in Table 1. One mL of blood was collected from the wing (cephalic) vein using a 25G needle and syringe, and immediately placed in a serum tube (1.3 mL BD Serum Tube®). Blood was allowed to clot for 6–24 hours prior to centrifugation and separation of serum and packed haemocytes. Urogenital (female bats), preputial (male bats), nasal, oral and rectal swabs (551C Copan® Minitip Flocked Swab) were collected, immediately placed in 500 μL of phosphate buffered saline (PBS), and kept chilled on ice prior to sample processing. Urine was collected by gentle trans-abdominal palpation of the bladder, and samples retained on ice prior to processing. Anaesthetised flying-foxes were assessed for haemostasis at the venepuncture site prior to being placed back in a pillow case, and their immediate post-anaesthetic recovery period monitored until they regained consciousness. At sampling locations where capture events extended over multiple days, flying-foxes had their toe-claws marked with an acrylic lacquer prior to release to enable identification of short-term re-captures. Flying-foxes were released at their point of capture after a minimum additional ½ hour recovery period. At the Boonah roost, *P*. *alecto* were preferentially targeted to address the objectives of the related longitudinal study. From December 2013, where the total daily capture limit was not met by *P*. *alecto* in any capture event, the balance was made up with *P*. *poliocephalus* and *P*. *scapulatus* selected to better balance overall sex and age classes.

**Table 1:** Classification of flying-fox demographic, morphometric and reproductive variables.

Variable	Description
Species	Black flying-fox (*P*. *alecto*)
	Grey-headed flying-fox (*P*. *poliocephalus*)
	Little red flying-fox (*P*. *scapulatus*)
Sex	Male
	Female
Age^1^	Juvenile
	Sub-adult
	Adult
Body condition score^2^	1: Poor condition
	2: Less than Fair condition
	3: Fair condition
	4: Greater than Fair condition
	5: Good condition
Weight	Body weight (g)
Forearm	Forearm length (mm)
Reproductive status^3^	Pregnant
	Dependent pup
	Lactating

^1^ Adult males distinguished from sub-adult males on the basis of fully developed penis and testes. Adult females distinguished from sub-adult females on the basis of worn, elongated nipples, indicating that they had suckled at least once in their lifetime. Females that were palpably pregnant but with no evidence of worn, elongated nipples were classified as adults. Juveniles (< 12 months old) were classified on their smaller size and rudimentary development of sexual characteristics.

^2^ Assessed primarily through palpation of the pectoral muscle mass and associated prominence of the sternal carinum; quantified on a 5-point scale.

^3^ Pregnancy was assessed through gentle trans-abdominal palpation of a gravid uterus (early–mid-pregnancy) or foetal structures (late pregnancy). Dependent pup is in reference to an adult female flying-fox with a totally dependent pup attached, and indicating the early post-partum period. Lactation (in the absence of a dependent pup) was confirmed through gentle expression of milk from enlarged nipples, and indicated the later post-partum period associated with a semi-independent juvenile.

### Sample processing and testing

Fifty μL of urine, serum, urogenital, preputial, nasal, oral and rectal swab samples were directly added to 130 μL of lysis solution (MagMAX, Ambion, Texas, Cat AM8500) to inactivate virus particles and preserve RNA for PCR screening. Total nucleic acid was immediately extracted from urine, serum, urogenital and preputial swab samples in lysis buffer using a magnetic bead based nucleic acid extraction kit (MagMAX-96 viral RNA isolation system, Ambion, Texas, Cat AM1836-5) run on a magnetic particle handling system (Kingfisher KF-96, Thermo-Scientific, Finland) according to the manufacturer’s instructions. The preserved nasal, oral and rectal swab samples were held at 4°C for 24–48 hours, before being stored at -80°C prior to testing several weeks later.

Urine, serum, urogenital and preputial swabs were used as an initial screening panel for all individual flying-foxes. Subsequently, the full complement of samples was tested from all HeV-positive flying-foxes, including the packed haemocyte sample retained after separation of serum. We also tested a subset of all available samples from all three species to ensure that possible additional routes of HeV excretion (i.e. nasal, oral and rectal secretions) weren’t being overlooked in the initial screening approach. The practical outcome of this process was to test the full complement of samples from an essentially random selection of *P*. *alecto*, *P*. *poliocephalus* and *P*. *scapulatus* captured at multiple sampling locations and across multiple times.

The AgPath-ID One-Step RT-PCR Kit (Life Technologies) was used for qRT-PCR, with 5uL of nucleic acid extract added to 20 μl of mastermix. Forward and reverse primers and probe that target a 69 base pair region on the M gene were used, as described by Smith et al. (2001) [28]. Positive and negative controls were included in each run. Assays were run on a 7500 Fast Real-Time PCR System (Applied Biosystems) in standard mode for a total of 45 cycles in accordance with the manufacturer’s instructions for the mastermix. A negative result (not detected) was determined if no amplification occurred within 40 cycles (i.e. the Ct value was equal to or greater than 40) as per Smith et al. (2001) [28].

## Results

A total of 2840 individual flying-foxes were captured and sampled across 10 roost sites over the 28-month period (Table 2). Of these, 42 flying-foxes had HeV RNA detected in at least one sample and were classified as “HeV-positive” (Table 3). Forty flying-foxes had HeV RNA detected on the initial screening panel; the two additional HeV-positive animals were identified from the subset which had the full complement of samples tested (animal numbers 2 and 9 in Table 3, with detections on a nasal swab and packed haemocyte sample respectively).

**Table 2:** Details of 2840 flying-foxes captured and sampled in the eastern Australian states of Queensland (QLD) and New South Wales (NSW) between February 2012 and June 2014.

Roost location	State	BFF ^1^	GHFF^1^	LRFF^1^	Sampling period
Boonah^2^	QLD	1045	747	214	May 2012—June 2014
Toowoomba^2^	QLD	236	1		April 2013
Sydney^3^	NSW	80	408		March—May 2012
Gayndah^3^	QLD	4		10	February—March 2012
Charters Towers^3^	QLD	4			March 2012
Calliope^3^	QLD	8			July 2012
Parkinson^3^	QLD	2	12		August 2012
Loders Creek^3^	QLD	18			August 2012
Laidley^3^	QLD	13		30	November 2012
Duaringa^3^	QLD			8	May 2012
**Total**		**1410**	**1168**	**262**	

^1^BFF = Black flying-fox (*Pteropus alecto*); GHFF = Grey-headed flying-fox (*P*. *poliocephalus*); LRFF = Little red flying-fox (*P*. *scapulatus*).

^2^ Roosts shared with a structured longitudinal study investigating HeV infection and transmission dynamics in flying-foxes (reported elsewhere); site selection changed after first bi-monthly sampling event from Toowoomba to Boonah.

^3^ Roosts shared with a cross-sectional study series investigating the association between flying-fox roost disturbance and HeV infection dynamics [25].

**Table 3:** qRT-PCR Ct results on biological samples, and physiological characteristics of 42 HeV-positive Black flying-foxes (*P*. *alecto*)^1^.

Animal	Sampling event	Sex^2^	Age^2^	Repro. Status^2^	Urine	Urogenital^2^	Serum	PHemo^2^	Rectal	Nasal	Oral
1	Jun-13	F	A	G		≥ 40	**33.53**	**35.848**	**30.27**	≥ 40	≥ 40
2	Jun-13	M	A		≥ 40	≥ 40	≥ 40	≥ 40	≥ 40	**36.99**	≥ 40
3	Jun-13	F	SA		**28.76**	**23.26**	**37.25**	**39.545**	**36.34**	≥ 40	≥ 40
4	Jun-13	F	A		**35.58**	≥ 40	**37.12**	≥ 40	≥ 40	≥ 40	≥ 40
5	Jun-13	F	A	GL	**36.80**	≥ 40	**37.15**	≥ 40	≥ 40	≥ 40	≥ 40
6	Jun-13	F	A	G	**22.26**	**24.721**	**34.64**	**31.7**	**26.76**	**33.28**	**33.65**
7	Jun-13	F	A	G		**35.291**	**36.14**	**35.41**	≥ 40	≥ 40	≥ 40
8	Jun-13	M	A		**36.82**	≥ 40	≥ 40	≥ 40	≥ 40	≥ 40	≥ 40
9	Jul-13	M	A		≥ 40	≥ 40	≥ 40	**36.48**	≥ 40	≥ 40	≥ 40
10	Jul-13	F	A		**29.38**	≥ 40	≥ 40	≥ 40	≥ 40	≥ 40	≥ 40
11	Jul-13	M	A		**29.31**	**32.93**	≥ 40	≥ 40	≥ 40	≥ 40	≥ 40
12	Jul-13	F	A	G		**36.79**	≥ 40	≥ 40	≥ 40	≥ 40	≥ 40
13	Aug-13	M	SA		**35.39**	≥ 40	≥ 40	≥ 40	≥ 40	≥ 40	≥ 40
14	Aug-13	F	A	G		**37.72**	≥ 40	≥ 40	≥ 40	≥ 40	≥ 40
15	Aug-13	F	A	G	**36.31**	≥ 40	≥ 40	≥ 40	≥ 40	≥ 40	≥ 40
16	Aug-13	F	A	G		**36.89**	≥ 40	≥ 40	≥ 40	≥ 40	≥ 40
17	Dec-13	F	A	L		**36.88**	≥ 40	≥ 40	≥ 40	≥ 40	≥ 40
18	Dec-13	M	A		**36.75**		≥ 40	≥ 40	≥ 40	≥ 40	≥ 40
19	Dec-13	F	A	LP	**37.33**	≥ 40	≥ 40	≥ 40	≥ 40	≥ 40	≥ 40
20	Feb-14	M	A		**34.57**		≥ 40	≥ 40	≥ 40	≥ 40	≥ 40
21	Feb-14	M	SA		**30.44**		**37.07**	**39.92**	≥ 40	≥ 40	≥ 40
22	Apr-14	M	A		**33.95**		≥ 40	≥ 40	≥ 40	≥ 40	≥ 40
23	Apr-14	F	A			**37.3**	≥ 40	≥ 40	≥ 40	≥ 40	≥ 40
24	Apr-14	M	A		**36.91**		≥ 40	≥ 40	≥ 40	≥ 40	≥ 40
25	Apr-14	M	A		**28.62**		≥ 40	≥ 40	≥ 40	≥ 40	≥ 40
26	Apr-14	M	A		**31.06**		≥ 40	≥ 40	≥ 40	≥ 40	≥ 40
27	Apr-14	F	SA			≥ 40	**34.06**	**35.29**	≥ 40	≥ 40	≥ 40
28	Apr-14	F	SA			**31.41**	≥ 40	≥ 40	≥ 40	≥ 40	≥ 40
29	Apr-14	F	A		**35.86**	**37.12**	**35.31**	**37.65**	≥ 40	≥ 40	≥ 40
30	Apr-14	F	A	L	**32.55**	**36.18**	≥ 40	≥ 40	≥ 40	≥ 40	≥ 40
31	Apr-14	M	A			**33.78**	≥ 40	≥ 40	**22.84**	≥ 40	≥ 40
32	May-14	F	A	G	**31.69**	**36.41**	≥ 40	≥ 40	**36.43**	≥ 40	≥ 40
33	May-14	M	A		**30.59**		≥ 40	≥ 40	≥ 40	≥ 40	≥ 40
34	May-14	F	A	G	≥ 40	≥ 40	**35.55**	≥ 40	≥ 40	≥ 40	≥ 40
35	May-14	F	A		**29.27**	≥ 40	≥ 40	≥ 40	**36.75**	≥ 40	≥ 40
36	May-14	F	SA			**35.05**	**37.19**	**37.35**	≥ 40	**37.64**	**36.31**
37	May-14	F	A	G		**33.05**	≥ 40	≥ 40	≥ 40	≥ 40	≥ 40
38	May-14	F	A	G	**33.56**	≥ 40	≥ 40	≥ 40	≥ 40	≥ 40	≥ 40
39	May-14	M	A		**37.12**		≥ 40	≥ 40	≥ 40	≥ 40	≥ 40
40	Jun-14	F	A	G	**25.71**	**32.73**	≥ 40	**37.45**	**35.16**	≥ 40	≥ 40
41	Jun-14	F	SA			**27.31**	≥ 40	≥ 40	**30.43**	≥ 40	≥ 40
42	Jun-14	F	A	G	**30.43**		≥ 40	≥ 40	≥ 40	≥ 40	≥ 40

^1^All 42 HeV-positive flying-foxes in the study were Black flying-foxes (*P*. *alecto*) caught at the Boonah roost.

^2^M = Male; F = Female: A = Adult; SA = Sub adult; J = Juvenile: G = Gestation; L = Lactation; P = Pup attached: Urogenital = vulvovaginal swab in females and preputial swab in males; PHemo = Packed haemocytes.

Of the three flying-fox species sampled during this study, HeV RNA was detected only in *P*. *alecto*, and all 42 HeV-positive *P*. *alecto* were captured at Boonah between June 2013–2014. The majority of HeV-positive flying-foxes had HeV RNA detected in a single sample (26/42; 61.9%), while a smaller number of animals had HeV RNA detected in multiple samples (i.e. 2 samples: 7/42; 16.7%; 3-or-more samples: 9/42; 21.4%). One individual flying-fox had HeV RNA detected on the full panel of samples available for testing (animal number 6 in Table 3; Ct values ranging from 22.26 in urine to 34.64 in serum). The overall estimate of HeV prevalence (95% binomial confidence intervals) in *P*. *alecto* across the entire study was 3% (2–4%).

Of the 29 HeV-positive *P*. *alecto* from which a urine sample was successfully collected, 26 (i.e. 90%) had HeV RNA detected in their urine, at Ct values ranging from 22.26–37.33 (Table 4). HeV RNA was also detected in over half of the urogenital swabs (including preputial swabs from male flying-foxes) collected from HeV-positive animals, at Ct values ranging from 23.26–37.72. Just over a quarter of HeV-positive flying-foxes had HeV RNA detected in their serum, and there were also detections in rectal swabs (8/42; 19%). Nasal and oral swabs yielded relatively low numbers of detections in HeV-positive animals, and at higher average Ct values than urine, urogenital and rectal swabs.

**Table 4:** qRT-PCR status summary statistics on biological samples from 42 HeV-positive Black flying-foxes (*P*. *alecto*)^1^.

Sample type	Positive	Negative	n	% Positive	Min C_T_	Max C_T_	Range C_T_	Mean C_T_	SD C_T_
Serum	11	31	42	26	33.53	37.25	3.72	35.91	1.38
PHemo^2^	10	32	42	24	31.70	39.92	8.22	36.66	2.35
Urine	26	3	29^3^	90	22.26	37.33	15.07	32.56	3.98
Urogenital	18	14	32^4^	56	23.26	37.72	14.46	33.60	4.38
Rectal	8	34	42	19	22.84	36.75	13.91	31.87	5.18
Nasal	3	39	42	7	27.64	33.28	5.64	35.97	2.35
Oral	2	40	42	5	33.65	36.31	2.66	34.98	1.88

^1^All 42 HeV-positive flying-foxes in the study were Black flying-foxes (*P*. *alecto*) caught at the Boonah roost.

^2^PHemo = packed haemocytes.

^3^ Urine samples were collected from 29/42 confirmed positive black flying-foxes.

^4^ Urogenital samples were collected from 32/42 confirmed positive black flying-foxes.

Of the 4 sample-types selected for HeV screening, urine yielded the highest number and percentage of HeV-positive detections in *P*. *alecto* (26/689; 4%), with less frequent detections urogenital and preputial swabs (18/1116; 2%) and serum (11/1385; 1%) (Table 5). The 42 HeV-positive *P*. *alecto* returned a total of 78 positive samples, at an overall detection rate of approximately 1.76% across all samples tested in this species (78/4436). There were no detections in *P*. *poliocephalus* (n = 1168 animals; n = 2958 samples) or *P*. *scapulatus* (n = 262 animals; n = 1191 samples) (Table 5).

**Table 5:** qRT-PCR summary statistics on biological samples collected from the three surveyed Australian *Pteropus* species.

	*P*. *alecto*			*P*. *poliocephalus*			*P*. *scapulatus*		
Biological sample	Neg	Pos	Prevalence (95% CI)	Neg	Pos	Prevalence (95% CI)	Neg	Pos	Prevalence (95% CI)
Serum	1385	11	1 (0–1)	1164	0	0 (0–0)	248	0	0 (0–2)
PHemo^1^	51	10	16 (9–28)	8	0	0 (0–32)	NT	NT	NT
Urine	663	26	4 (3–5)	534	0	0 (0–1)	94	0	0 (0–4)
Urogenital	1116	18	2 (1–2)	660	0	0 (0–1)	193	0	0 (0–2)
Rectal	385	8	2 (1–4)	202	0	0 (0–2)	216	0	0 (0–2)
Nasal	378	3	1 (0–2)	200	0	0 (0–2)	220	0	0 (0–2)
Oral	380	2	1 (0–2)	200	0	0 (0–2)	220	0	0 (0–2)
**TOTAL**	4358	78	2 (1–2)	2958	0	0 (0–0)	1191	0	0 (0–0)

^1^Packed haemocytes

## Discussion

This study represents the single largest sampling of individual wild-caught flying-foxes for HeV surveillance in Australia, and more broadly, for henipavirus surveillance globally. While other studies have used serological tools as a proxy for infection status of individual flying-foxes [29–31] to estimate prevalence, we were primarily interested in determining the major routes of HeV excretion in naturally-infected flying-foxes. The overall 3% HeV detection rate in *P*. *alecto* is higher than the 0.8% HeV detection rate reported by Breed et al. (2013), who sampled 124 *P*. *alecto* in Australia (n = 64), Papua New Guinea (n = 15) and Indonesia (n = 45) and detected one HeV-positive animal, using the same qRT-PCR for detection of HeV RNA, and with a comparable selection of screening samples (serum, urine and oral swabs). The single HeV-positive *P*. *alecto* described by Breed et al. (2013) was a sub-adult male, with detection of HeV RNA in blood, urine and saliva [22]. The Ct values of 35.3, 37.6 and 39.9 reported for these respective samples are essentially comparable to the values seen in our study, although the oral swab at a Ct of 39.9 is at the limits of detection for the assay in question.

One point of difference between our study and Breed et al. (2013) which may contribute to the difference in crude prevalence estimates is the physical choice of swab; the patented flocked swabs utilised in our study are purportedly more sensitive for bacterial and viral diagnostics than the cotton-tipped swabs used by Breed at al. (2013) [32–34]. Our study also involved a considerably larger sample size, and included both opportunistic cross-sectional and structured longitudinal sampling of flying-fox roosts; the latter is of particular importance in terms of accurately estimating HeV infection prevalence, as any temporal variation in viral shedding is captured by the longitudinal study design. The clustering of detections at the Boonah roost is plausibly explained by these features, given the greater sampling intensity over time at this roost and the relative over-representation of black flying-foxes at this roost. Other as yet unidentified factors may also have played a role.

Our study confirms that urine is the most important route of HeV excretion in naturally infected flying-foxes [16–21], and thus should be considered a priority sample from a diagnostic or surveillance screening perspective. Collecting a urine sample from flying-foxes is problematic however, as many animals will void their bladder at various points throughout the capture, holding, and sampling period, making urine recovery rates variable. We collected urogenital swabs from all animals to assess the relative diagnostic sensitivity of swabs. While there were 11 animals that were positive on urogenital swabs in the absence of a urine sample, only seven of 16 animals for which both samples were available tested positive on both, indicating that urogenital swabs underestimate HeV infection and excretion by about 50%. There were no instances where an animal tested negative on a urine sample but positive on a urogenital swab. These collective results suggest that the detection of HeV on a urogenital swab likely reflects the harvesting of residual HeV-positive urine within the lumen of the urogenital tract, rather than the detection of viral RNA associated with urogenital mucosal cells per se.

HeV-positive urine samples had comparable mean and minimum Ct values to HeV-positive urogenital swabs and rectal swabs. Mean and minimum Ct values in oral and nasal swabs were higher, indicating relatively lower levels of viral RNA associated with these portals. Although virus isolation was not attempted on any of the HeV-positive samples collected in this study, the use of Ct values as a semi-quantitative proxy for likely ‘viral load’ (or ‘intensity’ of viral shedding) is considered reasonable (see [9, 35, 36] for recent viral and bacterial examples, including HeV in experimentally-infected *P*. *alecto*). Given the successful isolation of HeV from pooled urine samples collected beneath Australian flying-fox roosts [17], and from an experimentally-infected *P*. *alecto* [9], it is reasonable to assume that viral RNA detected in the urine of naturally-infected flying-foxes can represent viable virus. The lowest Ct values observed in this study across a range of HeV-positive biological samples (around 22–23 units) are considerably lower than those observed in experimentally-infected *P*. *alecto* (28–29 units in select throat swabs and urine samples) [9]. Halpin et al. (2011) interpreted the low viral titres observed in these experimentally-infected bats as constraining the opportunity for equine spillover [9]. We suggest that the low Ct values observed in naturally-infected *P*. *alecto* in this study indicate that free-living flying-foxes are indeed capable of excreting potentially infectious viral loads. While previous virus isolation attempts from pooled flying-fox urine samples collected under-roost showed no apparent correlation with lower Ct values [17], we contend that lower Ct values in individual flying-fox urine samples are highly indicative of individual *P*. *alecto* shedding large amounts of potentially infectious virus in this excretory medium. In contrast to pooled urine samples, interpretation of the Ct value of individual urine samples is not complicated by pooling, samples are collected directly, and samples are immediately placed on ice. Future studies that pursue virus isolation from such samples would readily establish any correlation, although the BSL-4 pathogen status of HeV imposes significant logistical constraints to undertaking virus isolation experiments.

With respect to alternative routes of HeV excretion, the relatively limited number of detections on rectal swabs suggests that while faeces may potentially represent a source of infection to both flying-foxes and horses, it is much less significant a source than urine. This observation is consistent with relative detections in flying-fox urine and faeces collected under roosting flying-foxes [26]. There were fewer still detections on oral and nasal swabs, suggesting that oro-nasal secretions may be relatively unimportant as a mode of transmission. Additionally, we cannot rule out the possibility of urine contamination of both rectal and nasal swabs, which would effectively render any detection via these samples “false positives”. Urine contamination of female rectal swabs is particularly problematic given the close anatomical arrangement of the urogenital opening and the anus, and any urination event (including manual expression of the bladder) has the potential to result in urinary contamination of the perineum, with subsequent transfer of urine to rectal swabs likely. Urine contamination of nasal swabs is possible when flying-foxes urinate in their holding pillow-case; the animal’s head is then in direct apposition to the urine-soaked portion of the pillow-case, and urine contamination around the planum nasale is commonly observed. Despite these potential limitations in interpreting positive detections on rectal and nasal swabs, we did observe two instances where rectal and nasal swabs were positive in conjunction with confirmed negative urine and urine/urogenital swabs respectively (see animals 1 and 2 in Table 3), indicating the fundamental veracity of these routes of excretion. Oral swab-positive animals (animals 6 and 36 in Table 3) were observed only with concurrent HeV RNA detections across multiple samples, and while animals potentially shedding infectious virus from multiple excretory routes may provide some support for the “supershedder” hypothesis [13, 24], the small numbers observed ultimately preclude meaningful interpretation. The culture of live virus from HeV-positive nasal, oral and rectal swabs would clarify the relative importance of these routes in the transmission of HeV between flying-foxes, and from flying-foxes to horses.

The detection of HeV RNA in serum of naturally-infected *P*. *alecto* is of particular interest from a disease pathogenesis perspective, as it provides at least initial support for existing disease models that suggest localised viral infection at the point of inoculation (e.g. oropharyngeal mucosal replication) closely followed by systemic (i.e. haematogenous) spread to internal organs (e.g. lung, spleen, kidneys) [3, 8, 9, 37]. While the detection of HeV RNA in serum does not conclusively demonstrate an active viraemic phase, the presence of viable virus in blood is a necessary pre-condition for haematogenous dissemination of HeV to target organs. Culture of live virus from serum, packed haemocytes or (ideally) separated red and white blood cell components would facilitate greater understanding of how HeV spreads systemically within its natural flying-fox host. Berhane et al. (2008) have demonstrated in porcine models that the closely-related Nipah virus (NiV) is able to replicate in peripheral blood mononuclear cells (PMBCs), which facilitates both haematogenous spread and possible host immunosuppression due to viral replication in these particular immune cells [38].

Latent HeV infection interspersed with periods of recrudescence has also been hypothesised as a potentially important mechanism in maintaining persistent infection at both the individual bat and flying-fox population level [13, 39], and detection of HeV RNA in serum is at least consistent with the possible reactivation of a latent infection and haematogenous dissemination of virus to tissue sites where viral shedding can occur. A recent study identified the spleen as a possible site of latent HeV-infection in naturally-infected flying-foxes [37], and one plausible recrudescent pathway could involve haematogenous spread of virus from the spleen to kidney or bladder tissues, facilitating a patent viral infection in urine. While the small number of HeV RNA detections in serum precludes statistical significance testing, it is interesting to note that 10/11 (~91%) serum-positive animals were female. Additionally, all detections in female *P*. *alecto* clustered in April–June, which coincides with early to mid-gestation in this species (and half of all serum-positive females were indeed palpably pregnant at the time of sampling). Pregnancy has previously been recognised as a risk-factor for HeV infection in *P*. *scapulatus* and *P*. *conspicillatus*, using HeV serology as a proxy for infection status [29, 30], and the apparent association between HeV RNA detection in serum and pregnancy may be consistent with a recrudescent event in this particular demographic cohort. Halpin et al. (2011) also had an apparent sex-bias in relation to HeV RNA detections in blood from experimentally-infected *P*. *alecto* (4/5 blood-positive animals were female), although only one of these females was pregnant during the experimental period [9]. Alternatively, while the viraemic phase is generally presumed to be short in henipavirus infections in flying-foxes [8, 40], temporal or sex-related factors leading to differential viraemic periods could also explain an apparent demographic clustering of serum-positive females coinciding with early to mid-gestation.

The complete lack of detections in *P*. *poliocephalus* and *P*. *scapulatus* adds to a growing body of evidence suggesting that *P*. *alecto* and *P*. *conspicillatus* are the main reservoir host species for HeV [37, 41]. It could be argued that the lack of detections in *P*. *scapulatus* in this study may simply reflect the relatively limited, non-targeted sampling effort in this species (although n = 262, while modest compared to the *P*. *alecto* and *P*. *poliocephalus* samples, is substantial nonetheless), or alternatively, the narrow spatiotemporal sampling of *P*. *scapulatus* given its extensive geographic occurrence and highly nomadic nature [42]. Yet, with pregnancy previously associated with serological evidence of HeV infection in *P*. *scapulatus* [30], we were unable to detect any evidence of viral shedding in 83 adult females sampled at time points coincident with early-mid gestation for this species (Oct–Feb), including 43 confirmed pregnant animals. The complete absence of HeV detections in this higher infection risk cohort strengthens our contention that *P*. *scapulatus* is not a primary reservoir host.

Interpretation of the negative HeV data for P. *poliocephalus* is unambiguous given the predominant structured temporal surveillance at two disparate locations, and the large sample size (n = 1168). At the Boonah roost, which yielded the majority of the *P*. *poliocephalus* samples, all 42 HeV detections were made in co-roosting *P*. *alecto* in the same time period. At the Sydney Royal Botanic Gardens roost, the March–May 2012 sampling period encompassed breeding and early gestation in *P*. *poliocephalus* [43]. The lack of detections in this species at either location across various putative “high-risk” periods for HeV infection in flying-foxes [13, 29, 30, 44] strongly suggests that *P*. *poliocephalus* has a significantly lower infection rate than *P*. *alecto*. Our findings in relation to both *P*. *scapulatus* and *P*. *poliocephalus* are consistent with several other recent studies employing different sampling methodologies [16, 25], and provide additional evidence that HeV infection rates are low in these two species.

In terms of possible viral transmission pathways between flying-foxes, and from flying-foxes to horses, the results of this study provide new empirical evidence on the most likely sources of HeV exposure for both flying-foxes and horses. While failure to isolate HeV from nasal, oral, rectal and urinary samples from experimentally-infected *P*. *poliocephalus* has previously led authors to conclude that these potential excretory routes may be of little importance [8], a more recent appraisal of the evidence argues that early experimental infection models were likely flawed [37, 44]. The historical focus on the importance of infectious birthing material as a possible source of infection to horses [3, 8] has also been tempered by serological studies suggesting vertical transmission in flying-foxes is unlikely to be the predominant form of transmission [29, 30], and Goldspink et al. (2015) support this contention through an absence of HeV RNA detection in placental and uterine tissues from all four species of Australian mainland flying-fox [37]. The routine detection of HeV (and other paramyxoviruses) in pooled flying-fox urine samples [16–18, 21, 25] highlights the importance of this excretory medium in naturally-infected flying-foxes, and our study confirms that urine is the most significant route of HeV excretion in wild-caught *P*. *alecto*. Collectively these results suggest that flying-fox transmission likely revolves around direct contact with infectious urine of conspecifics (or possibly cross-species transmission in multi-species roosts). Hypothesised indirect transmission pathways facilitating spillover to horses via urinary contamination of pasture, feed and water sources remain plausible [44], although ingestion of contaminated ‘spats’ seems less likely given the low detection rates in oral swabs (notwithstanding the possibility that spats could be contaminated with infectious urine). The role of direct transmission pathways from flying-foxes to horses perhaps deserves greater attention. Our routine observation of the abundance of flying-fox urine, faeces and food debris underneath trees in which they are feeding suggests that horses underneath such trees have a high likelihood of direct physical contamination with potentially infectious material. Equine behavioural studies confirm that horses are essentially crepuscular in terms of their feeding activity, and while there is evident seasonal variation, horses in general spend more time resting at night than foraging [45, 46]. This infers that horses are more likely to be resting than grazing or browsing when flying-foxes are most actively foraging, making direct inoculation of infectious urine across oronasal or conjunctival surfaces potentially more plausible than ingestion. Both Field et al (2012) and Smith et al (2014) have previously alluded to the possibility of direct transmission [41, 44], and Field et al 2015 [47] elaborate the potential. A recent modelling paper reiterates the prospect [48]. Additional behavioural ecology studies on flying-fox and equine activities that influence potential contact rates at the bat-horse interface would further inform modes of transmission between flying-foxes and horses.

## Conclusion

We sought to identify the major routes of HeV excretion in naturally infected flying-foxes, and secondarily, to identify between-species variation in excretion prevalence. We captured free-living flying-foxes over space and time, and collected and screened multiple samples from each individual using a molecular approach to identify HeV RNA. The rate of detection, and the amount of viral RNA was highest in urine samples (>serum, packed haemocytes >faecal >nasal >oral), identifying urine as the most plausible source of infection for flying-foxes and for horses. Detection in a urine sample was more efficient than detection in urogenital swabs, identifying the former as the preferred diagnostic sample. The detection of HeV RNA in serum is consistent with haematogenous spread, and with hypothesised latency and recrudesence in flying-foxes. The lack of detections in *P*. *poliocephalus* and *P*. *scapulatus* strongly suggest these species are epidemiologically less important than *P*. *alecto*. The study is unprecedented in terms of our individual animal approach, our large sample size, and our use of a molecular assay to directly determine infection status. These features provide a high level of confidence in the veracity of our findings, and a sound basis from which to more precisely target equine risk mitigation strategies.
